# Using Mid-Upper Arm Circumference to End Treatment of Severe Acute Malnutrition Leads to Higher Weight Gains in the Most Malnourished Children

**DOI:** 10.1371/journal.pone.0055404

**Published:** 2013-02-13

**Authors:** Nancy M. Dale, Mark Myatt, Claudine Prudhon, André Briend

**Affiliations:** 1 Department of International Health, University of Tampere Medical School, Tampere, Finland; 2 Médecins Sans Frontières, Geneva, Switzerland; 3 Brixton Health, Llawryglyn, Wales, United Kingdom; 4 Health and Nutrition Tracking Service, Geneva, Switzerland; Universidad Peruana Cayetano Heredia, Peru

## Abstract

**Objective:**

The World Health Organization recommends discharging children admitted to nutrition programs treating severe acute malnutrition, with a low mid-upper arm circumference (MUAC <115 mm) when weight gain is >15%. When this recommendation is followed, the most severely malnourished children receive a shorter treatment compared to children that are less severely malnourished. This study assesses whether using MUAC >125 mm as discharge criteria eliminates this effect.

**Methods and Findings:**

Data from 753 children cured from a Médecins Sans Frontières outpatient nutrition program in Gedaref, North Sudan were analyzed. MUAC >125 mm was used as discharge criteria. Length of stay and percent weight gain of children were compared in relation to nutritional status on admission. Children with low MUAC on admission had a longer duration of treatment (*p* = 0.000) and also a higher percent weight gain (*p* = 0.000) than children with higher MUAC. Similar results with weight-for-height z-scores categories were shown with both duration of treatment (*p* = 0.000) and percent weight gain (*p* = 0.000).

**Conclusion:**

This study shows that using MUAC as the discharge criteria eliminates the effect of shorter treatment in most severely malnourished children compared to least severely malnourished, as is observed with percent weight gain. The findings directly address the main concern that has been identified with the current WHO recommendation of using percent weight gain. MUAC could be used as discharge criteria, instead of percent weight gain, as having a longer duration of treatment and a higher percent weight gain for the most malnourished is highly desirable.

## Introduction

Nearly 20 million under-5 children worldwide suffer from severe acute malnutrition (SAM) which contributes to the death of an estimated 1 million children per year [1]_._ The commonly used approach of the community management of acute malnutrition model (CMAM) includes both inpatient care in stabilization centres (SC) and/or outpatient care in outpatient therapeutic programs (OTP) depending on the presence or absence of complications [1]–[3].

In 2009 the World Health Organization (WHO) and United Nations Children’s Fund (UNICEF) provided new guidance on the admission criteria for programs treating SAM advising to include children less than -3 z-scores from the median weight-for-height z-scores (WHZ) of the WHO 2006 growth standards or a mid-upper arm circumference (MUAC) of less than 115 mm, and/or oedema [4]. They reinforced the use of MUAC as an independent admission criteria that had been introduced in an earlier statement [1].

Since the establishment of CMAM programs many organizations have started using MUAC for admission into their community based nutrition programs [5]. A plethora of literature emphasizing the advantages of using MUAC have supported this decision including: superior identification of children at high risk of mortality; simplicity of training; ability to allow for high coverage, and adherence to the WHO/UNICEF joint statement of maintaining consistency between screening method and admission criteria to avoid rejected referrals [4]–[6]. Furthermore, the difficulties of taking accurate weight and height measurements compared to MUAC are well documented [7], [8] and reliable height measurements are not easily performed in the community. At most primary healthcare centres in developing countries, height boards are not present and are not part of essential supplies packs. Measuring height is also not covered in the Integrated Management of Childhood Illness syllabus [9], [10].

In addition to guidance on admission criteria for nutrition programs, the WHO and UNICEF 2009 Joint Statement also gave some guidance regarding discharge criteria in nutrition programs using MUAC on admission, recommending to discharge children after reaching a 15–20% weight gain [4]. This was done with the intention of removing the need of height measurement and avoiding the problem of children meeting MUAC admission criteria but having a WHZ above discharge criteria [5].

Using percent weight gain as discharge criteria has the disadvantage of requiring a smaller absolute weight gain to meet discharge criteria for children with the lowest initial weight, (i.e. the most severely malnourished children). This leads to shorter duration of treatment for the most malnourished children as weight gain is higher in the most wasted children receiving appropriate treatment [11]. This unfavourable consequence on treatment duration was documented by Médecins Sans Frontières (MSF) from a nutrition program in Burkina Faso [12]. Shorter treatment of the most severely malnourished is worrisome and needs to be addressed. Prolonged length of stay for some children also has considerable consequence to on-going government programs in resource scarce settings.

Based on the difficulties with the current recommendation of percent weight gain for discharge and on evidence that MUAC and weight respond to treatment in similar ways [13], a decision was made by MSF Switzerland to use MUAC as both admission and discharge criteria in an emergency nutrition program in Gedaref, North Sudan.

The objective of this paper is to evaluate the policy of using MUAC as discharge criteria in outpatient therapeutic programs and more specifically to see whether it eliminates the effect of shorter treatment duration in most severely malnourished children as observed with percent weight gain.

## Methods

### Ethics Statement

Written consent was not obtained from the caretakers for storing data used for this analysis in the MSF database as these data were collected as part of routine patient monitoring and program evaluation. Furthermore, these data were entered anonymously before being used for this analysis. Ethical approval for the study was given by the Ethical Review Board of Médecins Sans Frontières.

### Program Details

In July 2010 MSF Switzerland, in collaboration with the Ministry of Health, launched an emergency nutrition program in the localities Gala Alnahal and Al Guiresha in Gedaref, North Sudan. The total estimated population of children less than 5 years for the two localities was around 25 000. Gedaref is a state known normally for its strong crop production producing sorghum and sesame for the entire country. During the years of 2008–2010 Gedaref experienced poor rainfall and thus decreased crop production. As a result, there were a large number of small farmers and agricultural workers that experienced notable reduction of their income and food sources particularly among the lower socio economic groups who were already quite vulnerable to economic shocks.

The program treated malnourished children in a community-based therapeutic feeding program in 4 SCs and 77 OTPs. Children aged 6 to 59 months with MUAC <115 mm and/or mild oedema had an initial assessment by a physician or medical assistant for the presence of diarrhea, vomiting, anorexia, anemia (based on conjunctiva pallor), and fever. The children were also checked for malaria parasitemia using a rapid diagnostic test, specifically Paracheck Pf ®, and whether they had previously been immunized against measles. Children with good appetite and no severe medical complications (i.e., severe anemia shock, sepsis, severe dehydration, anorexia, severe oedema) were classified as having uncomplicated SAM and admitted to the OTP. Children in the OTP received Ready to Use Therapeutic Food as per WHO guidelines [1]. All children with MUAC <115 mm with severe medical complications and those requiring 24-hour close observation were hospitalised in an SC until they were stable enough to be transferred to an OTP, following the CMAM approach [2], [3]. Children requiring stabilization were referred to the nearest MSF SC with transportation provided by MSF.

All children in the program had their height, weight, MUAC and oedema checked at admission; weight, MUAC and oedema were checked at each visit; weight and MUAC were checked at discharge.

Pediatric height boards of standard design were used to measure height as per MSF nutritional guidelines [14] to the nearest millimetre and spring hanging scales (Salter Brecknell, Fairmount, Minnesota) were used to measure weight to the nearest 100 grams. Children were weighed naked or lightly clothed. Scales were reset to zero between each weighing. UNICEF MUAC bracelets were used to measure MUAC to the nearest millimetre. Most children were checked every 2 weeks except for some children who attended the 4 OTP sites located beside the 4 inpatient facilities who were seen every week.

The following criteria for discharge were required: MUAC >125 mm for 2 consecutive measurements plus stable weight or continuing weight gain; absence of bilateral oedema for 4 weeks; clinically well and with good appetite. MUAC cut off for discharge was set at 125 mm based on evidence that there is very low mortality in children with MUAC above 125 mm [5].

All children aged 6–59 months with a MUAC <115 mm on admission who were discharged as cured from the OTP were included in the study. The study was limited to children admitted directly to the OTP as duration of treatment and weight gain during the stabilisation phase is mainly influenced by associated medical complications and largely unrelated to response to therapeutic feeding. This also excluded from the analysis children with severe oedema who were treated initially in SC. These children receive a low-protein low-energy diet at the beginning of treatment which does not allow for new tissue synthesis and weight change during these initial days and is more related to body water elimination than to synthesis of new tissues [15].

The decision to use MUAC as discharge criteria was made by MSF for programmatic reasons. This analysis was carried out only with data routinely collected to monitor the program according to current MSF guidelines and thus individual written consent was not obtained. All data entered into the database was done anonymously.

### Data Analysis

**Figure 1:**
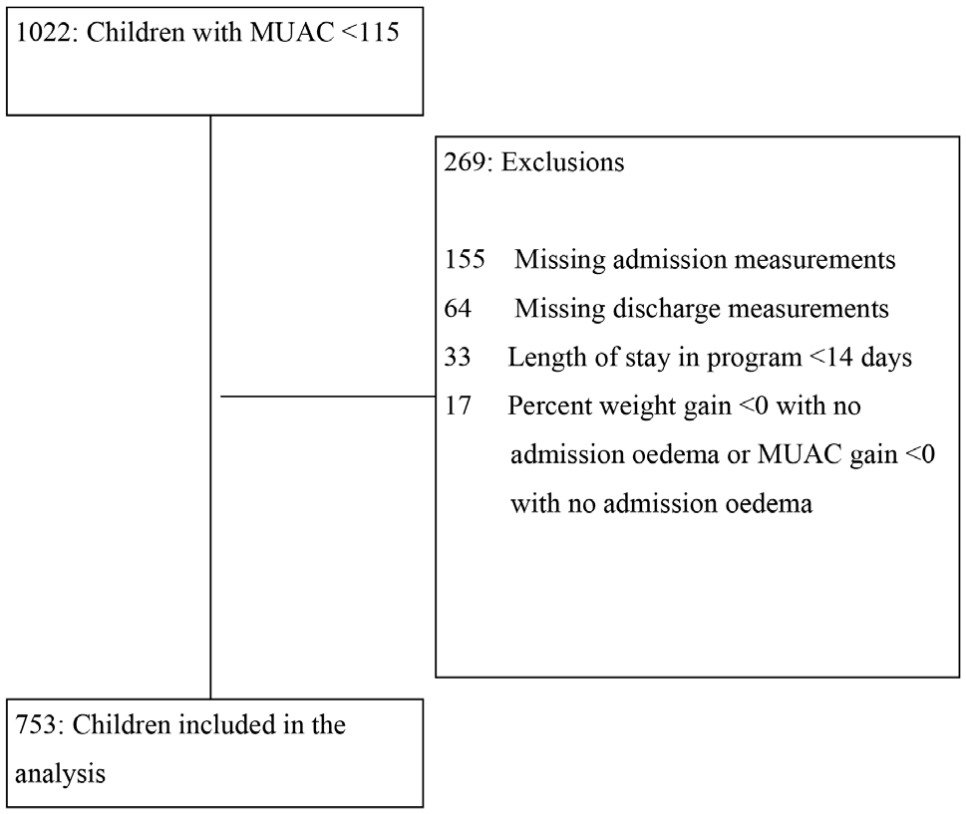
Flowchart of Outpatient Therapeutic Program children excluded from analysis.

Children were admitted into the program from July 12, 2010 to December 11, 2010, the last patient was discharged from the program on December 22, 2010. Anthropometric data of all children in the program were recorded in the study database. The initial number of children meeting the inclusion criterion (MUAC <115 mm) who were cured in the OTP was 1022. The following children were excluded from the study: i) missing admission data or discharge data; ii) a percent weight gain <0 or percent MUAC gain <0 with no oedema on admission; iii) a length of stay less than 14 days. The flowchart describes exclusion of these children from the study sample (Figure 1).

Children were classified into different categories based on admission MUAC and WHZ. Duration of treatment and percent weight gain were compared across these categories. Percent weight gain was also compared for different height categories, height being used as a proxy for age in this population where accurate ascertainment of age is difficult.

Statistical analysis was done by using MYSTAT (Stystat Software, Chicago, Illinois. 2008). WHZ was calculated using the WHO Anthro (version 3.2.2 January 2011) software. The Kruskal-Wallis non parametric test was used for comparisons of lengths of stay and percent weight gain by categories of MUAC at admission, height at admission, and WHZ at admission. This non-parametric procedure was used because it is robust to deviations from normality and because median length of stay is a more useful measure of the average length of treatment episodes than is the mean length of stay which can be influenced by extreme values from (e.g.) a handful of complicated cases. The null hypothesis for the Kruskal-Wallis test is that the populations from which the sample originates have the same median. Cochran’s test for linear trend was used to look at the association between ordered and contiguous MUAC categories and the proportion of children achieving a weight gain of at least 15%. The null hypothesis for Cochran’s test for linear trend tells us that the slope of a regression line across the proportions is zero.

## Results

**Figure 2:**
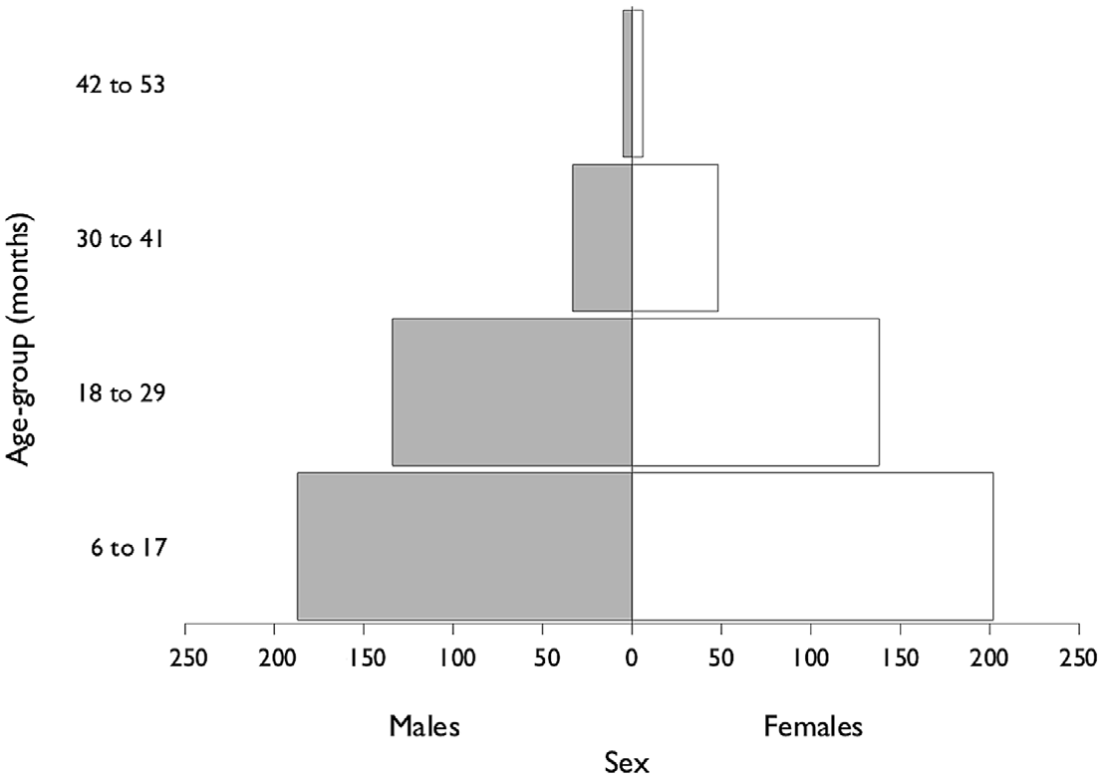
Population profile of the Outpatient Therapeutic Program.

753 children were included in the analysis. 52% of the children were female and the median age was 16 months. 88% of the children in the program were between 6 months and 29 months. The profile of the study population by age and sex is shown in Figure 2.

Outcomes of the children in the OTP were within SPHERE standards [16] with proportions of cured, defaulter and deaths being 82%, 15% and 1% respectively. The remaining 2% were referred to the main district general hospital for care beyond what was available from the MSF program.

**Figure 3:**
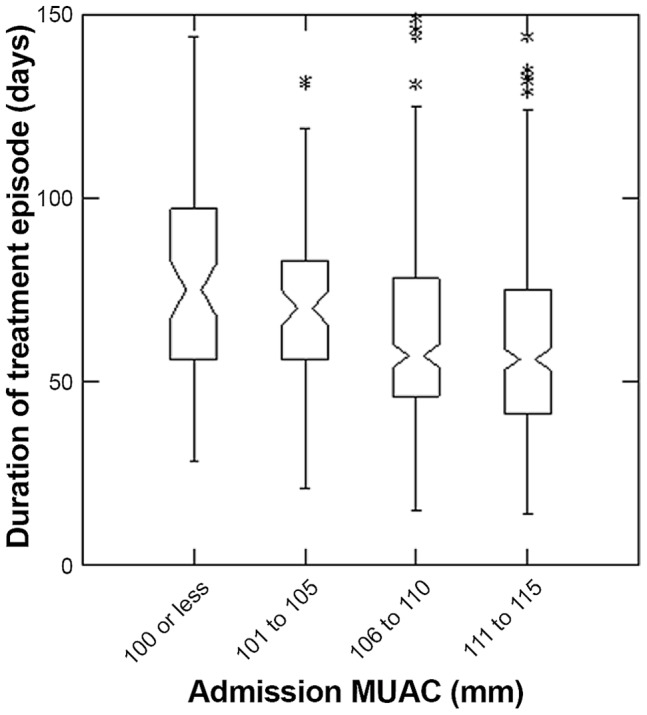
Length of Stay by categories of MUAC on admission to Outpatient Therapeutic Program. The box extends between the upper and lower quartiles, the line in the box marks the position of the median, the notches around the median show the extent of an approximate 95% confidence for the position of the median. The whiskers extend to 1.5 times the interquartile distance above and below the upper and lower quartiles, and the asterisks mark the positions of points more extreme than the range of values covered by the whiskers.

**Figure 4:**
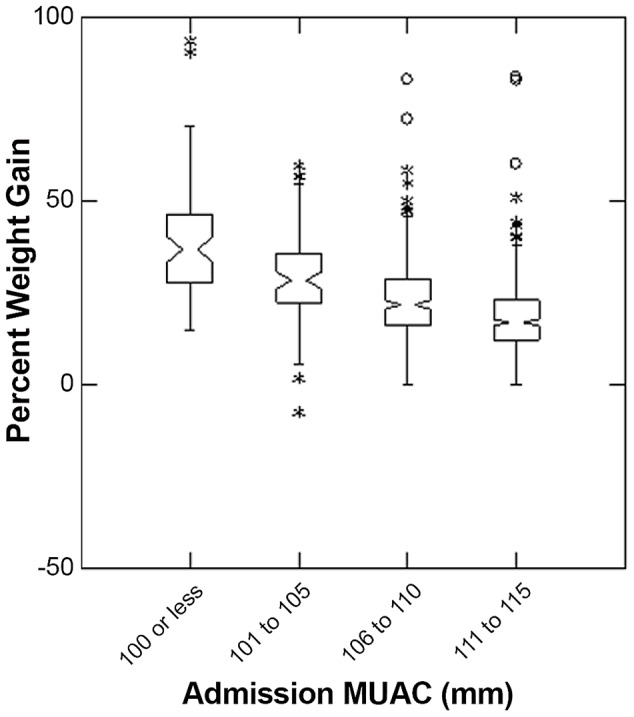
Percent Weight Gain by categories of MUAC on admission to Outpatient Therapeutic Program. The box extends between the upper and lower quartiles, the line in the box marks the position of the median, the notches around the median show the extent of an approximate 95% confidence for the position of the median. The whiskers extend to 1.5 times the interquartile distance above and below the upper and lower quartiles, and the asterisks mark the positions of points more extreme than the range of values covered by the whiskers.

**Figure 5:**
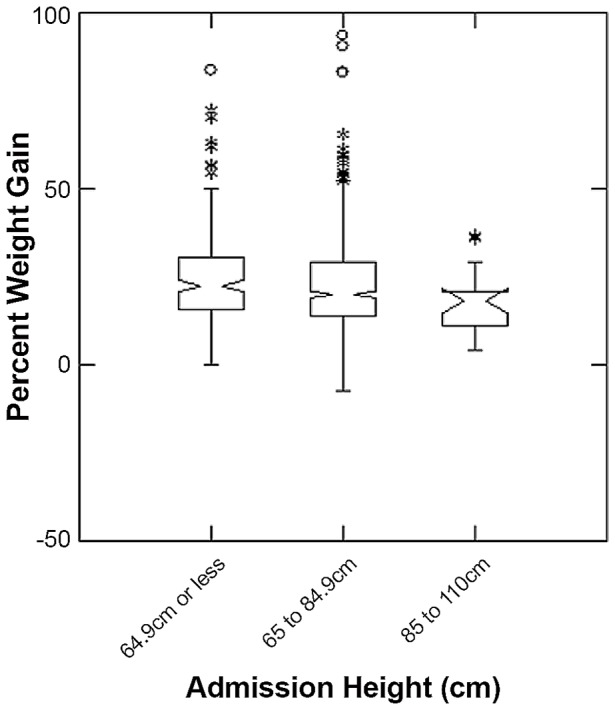
Percent Weight Gain for different categories of height on admission to Outpatient Therapeutic Program. The box extends between the upper and lower quartiles, the line in the box marks the position of the median, the notches around the median show the extent of an approximate 95% confidence for the position of the median. The whiskers extend to 1.5 times the interquartile distance above and below the upper and lower quartiles, and the asterisks mark the positions of points more extreme than the range of values covered by the whiskers.

The overall median length of stay of all children in the study was 60 days (inter-quartile range (IQR) = 43; 81). Children with lower MUAC at admission had longer durations of treatment (p<0.001 Kruskal-Wallis test), with median durations of treatment in the lowest MUAC group of 75 days (IQR = 56; 97) and highest MUAC group of 56 days (IQR = 41; 75). The overall percent weight gain of all children in the study was 21 (IQR = 14; 29). Children with low MUAC also had higher percent weight gain (p<0.001 Kruskal-Wallis test), with median percent weight gain of 37 in the lowest MUAC group (IQR = 28; 47) and 17 in the highest MUAC group (IQR 12; 23) (Figs. 3 and 4). Response to treatment was independent of height at admission (p>0.05 Kruskal-Wallis test) (Figure 5).

The majority of children in all MUAC categories gained more than 15% of their weight at admission, with the highest proportion among those with the lowest MUAC (Cochran’s test for linear trend = 64.120, p<0.001) (Table 1).

**Table 1:** Weight gain greater than 15% by MUAC category on admission to Outpatient Therapeutic Program.

MUAC category on admission (mm)	Total N	Weight gain greater than 15% N (%)
**Less than 100**	73	72 (99)
**101 to 105**	81	69 (85)
**106 to 110**	246	190 (77)
**111 to 115**	353	207 (59)
**Total**	753	538 (71)

**Figure 6:**
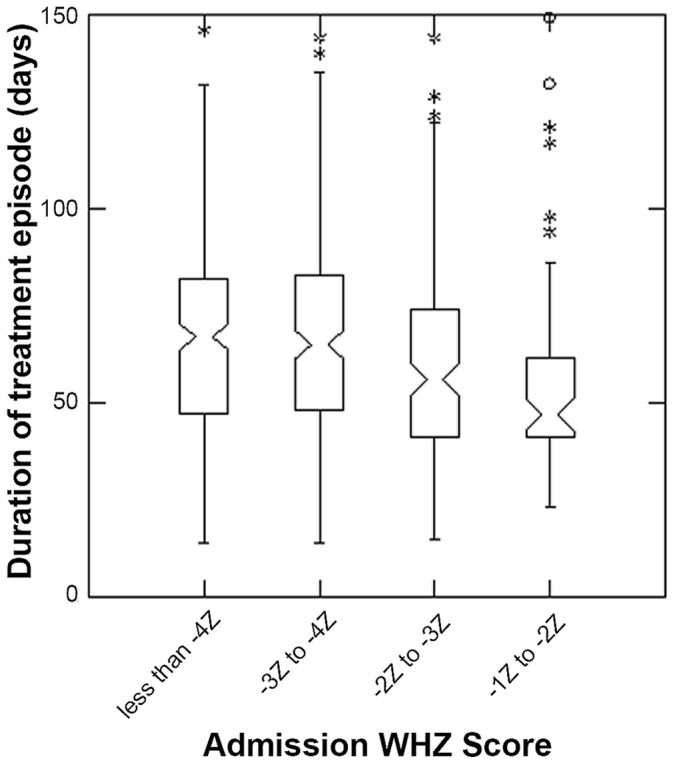
Length of Stay for different categories of WHZ on admission to Outpatient Therapeutic Program. The box extends between the upper and lower quartiles, the line in the box marks the position of the median, the notches around the median show the extent of an approximate 95% confidence for the position of the median. The whiskers extend to 1.5 times the interquartile distance above and below the upper and lower quartiles, and the asterisks mark the positions of points more extreme than the range of values covered by the whiskers; all children had mid-upper arm circumference <115 mm.

**Figure 7:**
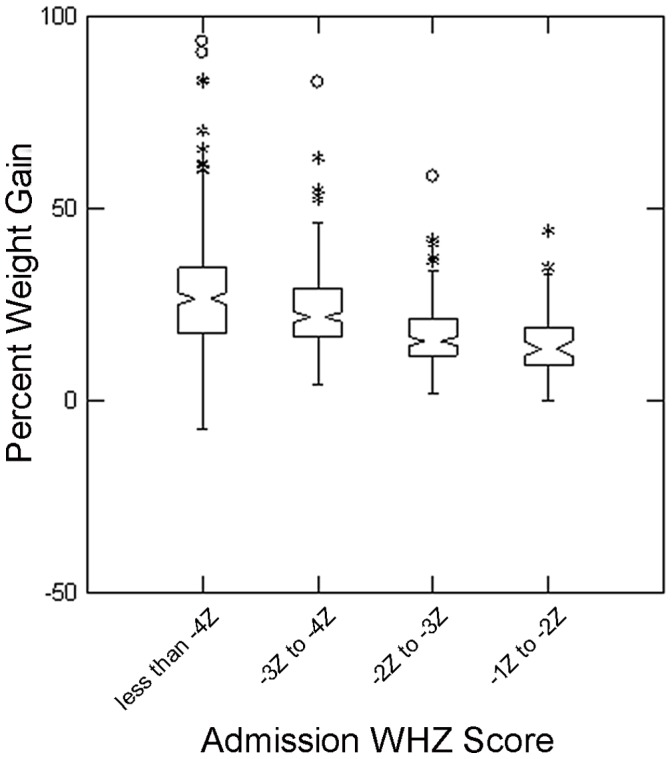
Percent Weight Gain for different categories of WHZ on admission to Outpatient Therapeutic Program. The box extends between the upper and lower quartiles, the line in the box marks the position of the median, the notches around the median show the extent of an approximate 95% confidence for the position of the median. The whiskers extend to 1.5 times the interquartile distance above and below the upper and lower quartiles, and the asterisks mark the positions of points more extreme than the range of values covered by the whiskers; all children had mid-upper arm circumference <115 mm.

Similar results for WHZ to those for MUAC categories on admission were found, with both duration of treatment (p<0.001 Kruskal-Wallis test) and percent weight gain (p<0.001 Kruskal-Wallis test) decreasing as WHZ categories increased (Figures 6 and 7).

## Discussion

This study shows that using MUAC as discharge criteria eliminates the effect of shorter treatment in most severely malnourished children and longer treatment for least severely malnourished, as observed with percent weight gain. The findings directly address the main concern that has been identified with using the current WHO recommendation of percent weight gain.

The study also shows that as a result of the longer treatment, the most severely malnourished children such as those with the lowest MUAC on admission achieve a higher percent weight gain than the recommended 15%. Consistent results were obtained when children were classified according to their weight-for-height z-scores. Again the length of stay is longer and percent weight gain is higher for the most severely malnourished.

One limitation to the study is the number of children who were excluded from the analysis. Most exclusions (81%) were due to missing admission and discharge measurements, mainly due to missing height data. These missing measurements were unavoidable since we were working with routine program data from an emergency nutrition program. They also occurred randomly and are unlikely to have biased our results. In this regard, admission MUACs were compared in the dataset prior to these exclusions and no significant differences were found from the final results so biases from the exclusions seem unlikely.

Since MUAC has been accepted as independent admission criteria for SAM treatment, criteria for discharge have remained debated. WHZ, the nutritional index which was previously recommended as discharge criteria for more than 20 years could not be recommended, as there is an imperfect match between MUAC and WHZ and some children may qualify for discharge shortly after admission or even on admission. For simplicity, it would be easier to use the same criteria for admission and discharge however MUAC has not been used so far as there is little data on response to treatment of this anthropometric measure. Percent weight gain was recommended, despite the expected undesirable effect of having shorter duration of treatment for the most malnourished children. Our results suggest that MUAC responds to treatment and that using MUAC as discharge criteria leads to an average similar percent weight gain to the 15–20% recommended by WHO. Our results also show that the duration of treatment and percent of weight gain are higher in the most malnourished children.

A formal cost comparison of using MUAC versus percent weight gain as discharge criteria in nutrition programs is beyond the scope of this paper. However we did look at the length of stay which is a major determinant of the cost of treatment. We compared this Gedaref program that used MUAC as discharge criteria with the 2009 MSF program in Burkina Faso that used 15 percent weight gain as their discharge criteria. The average lengths of stay in these programs for children admitted with a MUAC less than 115 were 64 [12] in Gedaref compared to 51 days in Burkina Faso. As the Burkina Faso program used the lower limit of the WHO recommended 15–20 percent weight gain for discharge [4] and as the Gedaref program was only able to see the children every 2 weeks due to the emergency context compared to every week in the Burkina Faso program (this extends the period for the two proof-of-cure measurements from two weeks to four weeks), the use of MUAC as discharge does not seem to increase the length of stay and thus the cost of the program. However, it is important to note that length of stay may vary from place to place and these findings have to be confirmed by other programmes using the same cut off for discharge.

We suggest using MUAC as discharge criteria, instead of a uniform percent weight gain, as having a longer duration of treatment and a higher percent weight gain for the most malnourished is highly desirable. Furthermore, the feasibility of using MUAC is less complicated than using percent weight gain and more transparent for mothers to understand.

Our study suggested that using MUAC >125 mm was feasible and led to acceptable durations of treatment and weight gains in our setting. It is not possible from our data to assess whether this discharge criteria was optimal or whether it would be applicable to other settings. A follow-up of children discharged with a MUAC >125 mm with close monitoring of relapse and risk factors would be useful to confirm its validity. In addition, the validity of this cut-off should be confirmed in other settings.
